# Rosiglitazone Suppresses Calcium Oxalate Crystal Binding and Oxalate-Induced Oxidative Stress in Renal Epithelial Cells by Promoting PPAR-*γ* Activation and Subsequent Regulation of TGF-*β*1 and HGF Expression

**DOI:** 10.1155/2019/4826525

**Published:** 2019-11-12

**Authors:** Ya-Dong Liu, Shi-Liang Yu, Rui Wang, Jian-Nan Liu, Yin-Shan Jin, Yi-Fu Li, Rui-Hua An

**Affiliations:** Department of Urology, The First Affiliated Hospital of Harbin Medical University, No. 23 You Zheng Street, Harbin, 150001 Heilongjiang, China

## Abstract

Peroxisome proliferator-activated receptor- (PPAR-) *γ* is a ligand-dependent transcription factor, and it has become evident that PPAR-*γ* agonists have renoprotective effects, but their influence and mechanism during the development of calcium oxalate (CaOx) nephrolithiasis remain unknown. Rosiglitazone (RSG) was used as a representative PPAR-*γ* agonist in our experiments. The expression of transforming growth factor-*β*1 (TGF-*β*1), hepatocyte growth factor (HGF), c-Met, p-Met, PPAR-*γ*, p-PPAR-*γ* (Ser112), Smad2, Smad3, pSmad2/3, and Smad7 was examined in oxalate-treated Madin-Darby canine kidney (MDCK) cells and a stone-forming rat model. A CCK-8 assay was used to evaluate the effects of RSG on cell viability. In addition, intracellular reactive oxygen species (ROS) levels were monitored, and lipid peroxidation in renal tissue was detected according to superoxide dismutase and malondialdehyde levels. Moreover, the location and extent of CaOx crystal deposition were evaluated by Pizzolato staining. Our results showed that, both *in vitro* and *in vivo*, oxalate impaired PPAR-*γ* expression and phosphorylation, and then accumulative ROS production was observed, accompanied by enhanced TGF-*β*1 and reduced HGF. These phenomena could be reversed by the addition of RSG. RSG also promoted cell viability and proliferation and decreased oxidative stress damage and CaOx crystal deposition. However, these protective effects of RSG were abrogated by the PPAR-*γ*-specific inhibitor GW9662. Our results revealed that the reduction of PPAR-*γ* activity played a critical role in oxalate-induced ROS damage and CaOx stone formation. RSG can regulate TGF-*β*1 and HGF/c-Met through PPAR-*γ* to exert antioxidant effects against hyperoxaluria and alleviate crystal deposition. Therefore, PPAR-*γ* agonists may be expected to be a novel therapy for nephrolithiasis, and this effect is related to PPAR-*γ*-dependent suppression of oxidative stress.

## 1. Introduction

Emerging data have shown that the incidence and prevalence of nephrolithiasis are increasing remarkably [1]. Approximately 70% of human kidney stones are primarily composed of calcium oxalate (CaOx) [2]. Previous studies have shown that a high concentration of oxalate leads to renal tubular epithelial cell injury and contributes considerably to the deposition and progression of CaOx crystals [3]. A growing body of evidence suggests that increased CaOx crystal adhesion to and aggregation in renal tubular cells are associated with the overproduction of reactive oxygen species (ROS) [4]. ROS result in lipid peroxidation of the cellular membranes and serious renal tissue injuries, presumably through oxidative stress [5]. Therefore, antioxidant therapy can attenuate damage caused by renal oxidative stress and prevent CaOx deposition.

Peroxisome proliferator-activated receptor-*γ* (PPAR-*γ*), a nuclear hormone receptor, plays central roles in cellular proliferation, morphogenesis, and inflammation as well as the maintenance of cell metabolic homeostasis [5–7]. PPAR-*γ* has also been reported to be highly expressed in renal tissues, which are mainly expressed in distal tubules, the inner medullary collecting ducts, and the thick ascending limb of Henle's loop [8, 9]. It modulates gene expression by binding to peroxisome proliferator response element (PPRE) sites and affects the transcription of downstream target genes [10]. Li et al. demonstrated that the PPAR-*γ* agonist 15-deoxy-*Δ*12,14-prostaglandin J2 (15d-PGJ2) can prevent CaOx stone formation both *in vivo* and *in vitro* [11]. However, the mechanism of PPAR-*γ* signaling in oxalate-induced tubular cell injury requires further investigation.

It is well known that PPAR-*γ* agonists have antioxidant and antifibrosis functions [12–15]. Transforming growth factor-*β*1 (TGF-*β*1), as a cytokine with multiple biological functions, plays a prominent role in a variety of kidney diseases, such as glomerulonephritis, renal interstitial fibrosis, and nephrolithiasis [16, 17]. Oxalate-induced ROS production is considered to be the result of TGF-*β*1 activation [17]. Recently, multiple studies have suggested that PPAR-*γ* agonists can exert a therapeutic effect on kidney disease by inhibiting TGF-*β*1 signal transduction [11]. PPAR-*γ* agonists can inhibit TGF-*β*1 mRNA and protein expression [7]. However, it has been confirmed that PPAR-*γ* binds to the putative PPRE in the promoter region of the hepatocyte growth factor (HGF) gene after ligand stimulation and leads to increased HGF gene transcription, mRNA expression, and protein secretion. HGF has been considered a downstream effector of PPAR-*γ* agonists, and some researchers suggest that the antifibrotic activity of PPAR-*γ* agonists is mediated primarily by HGF expression [18]. The HGF/c-Met signal transduction pathway is considered a key regulator of cellular oxidative stress, and exacerbating the activity of this pathway can improve cellular antioxidant capacity [19]. As Imamura et al. reported, the administration of exogenous HGF can attenuate cell-crystal interactions and crystal precipitation in hyperoxaluric rats [20]. Therefore, we hypothesized that PPAR-*γ* serves as a regulator of renal tubular epithelial cell redox status by influencing TGF-*β*1 and HGF. However, there are few studies on the mechanism and effect of PPAR-*γ* agonists in CaOx models.

PPAR-*γ* agonists include endogenous ligands such as 15d-PGJ2 and synthetic ligands, such as thiazolidinediones (TZDs) [21, 22]. However, the mechanism of the antilithogenic effects induced by PPAR-*γ* agonists remains unclear. TZDs are highly potent PPAR-*γ* agonists and include rosiglitazone (RSG), pioglitazone (PGZ), and troglitazone (TGZ) [20–22]. RSG is a typical representative PPAR-*γ* agonist and has recently been reported to exert antioxidant effects via a PPAR-*γ*-dependent mechanism [12, 22]. In this study, we used *in vitro* and *in vivo* experiments to investigate the alterations in PPAR-*γ* and its downstream effects in a hyperoxaluric environment and to determine whether RSG exerts renal protective effects by regulating TGF-*β*1 and HGF through a PPAR-*γ*-dependent signaling pathway.

## 2. Materials and Methods

### 2.1. Reagents

A canine renal distal tubular epithelium cell line (MDCK) was purchased from the American Type Culture Collection (Rockville, USA). RSG, GW9662, and PHA665752 were obtained from Sigma-Aldrich (St. Louis, MO, USA). The following primary antibodies were used: p-Met, Smad7, TGF-*β*1, and PPAR-*γ* (Santa Cruz, USA); HGF (Abcam, USA); p-PPAR-*γ* (Ser112) (Bioss, China); c-Met (Proteintech, China); Smad2, Smad3, p-Smad2 3, and Lamin B (Wanleibio, China); and GAPDH (Zhongshan Golden Bridge Bio Co., Ltd., China). The secondary antibodies, including HRP-conjugated AffiniPure goat anti-rabbit IgG and HRP-conjugated AffiniPure goat anti-mouse IgG, were purchased from Zhongshan Golden Bridge Bio Co., Ltd. (Beijing, China). A Nuclear and Cytoplasmic Protein Extraction Kit was acquired from Beyotime Institute of Biotechnology (Shanghai, China).

### 2.2. Cell Culture

MDCK (CCL34, passages 53 to 90) was cultured in Dulbecco's modified Eagle's medium (DMEM) supplemented with 10% fetal bovine serum (FBS; Life Technologies) and antibiotics (100 U/mL penicillin and 100 *μ*g/mL streptomycin; Life Technologies, Carlsbad, CA, USA) and kept in a humidified atmosphere with 5% CO_2_ at 37°C. Cells were grown to 80%-90% confluence and subcultured by treatment with 0.25% trypsin and 1 mM ethylenediaminetetraacetic acid (Beyotime Institute of Biotechnology, Shanghai, China). Cells were then coincubated with oxalate (0.5 mM; Sigma-Aldrich), RSG, or GW9662 for 1, 2, and 4 h at 37°C in calcium ion-free minimum essential medium. All experiments were done in triplicate.

### 2.3. Animal Model and Experimental Design

Sixty male Sprague-Dawley rats weighing 150-200 g were housed in clean plastic cages under controlled temperature (22-25°C), humidity, and light (12 h light/dark cycle) with free access to food and water. The use of animals and the experimental protocols were reviewed and approved by the Institutional Animal Care and Use Committee, and animals were treated in accordance with the National Institutes of Health Guidelines of Laboratory Animal Care and Use. After a week of adaptation to our experimental animal environment, 60 rats were randomly divided into five groups as follows: (1) control group (standard diet), (2) ethylene glycol (EG) group (calcium oxalate crystal caused by 0.75% EG administration), (3) RSG group (administered with EG daily and treated with RSG via intragastric administration), (4) GW9662 group (treated with GW9662 via gastric perfusion), and (5) RSG+GW9662 group (administered with EG and treated with RSG and GW9662 via intragastric administration). Each group consisted of two subgroups of five animals that were treated as above for 7 or 14 days, respectively. On days 7 and 14 after treatment with EG, urine samples were collected for 24 h period from rats in the metabolic cages and then both rats were anaesthetized. Before sacrificing, the blood was collected from the inferior vena cava for biochemical analysis. The right kidney of each rat was fixed and stored in 4% paraformaldehyde solution. The left kidneys were frozen immediately in liquid nitrogen and then stored at -80°C before using.

### 2.4. Cell Viability Assay

To evaluate the effects of RSG on cell viability, a CCK-8 assay was used. MDCK cells were seeded into 96-well culture plates at a density of 8000 cells per well and grown for 24 h. Cells were cotreated with various concentrations of RSG (0, 2, 4, 8, 16, 32, and 64 *μ*mol/L) and oxalate (0.5 mM) in DMEM medium for 1, 2, and 4 h. At each time point, 10 *μ*L of CCK-8 (Dojindo Laboratories, Japan) solutions was added to each well and mixtures were incubated at 37°C in an incubator for additional 1 h. The absorbance at 450 nm was read using a microplate reader (ELx808, BioTek Instruments, Inc., Winooski, VT, USA).

### 2.5. Measurement of ROS Generation

The level of intracellular ROS induced by oxalate in the presence or absence of RSG and GW9662 was monitored using a dichlorofluorescein diacetate (DCFH-DA) cell-permeant probe (Beyotime Institute of Biotechnology, Shanghai, China). Briefly, the cells from different groups were collected and cultured with 10 *μ*mol/L DCFH-DA at 37°C for 20 min and then rinsed twice with serum-free DMEM to remove the extracellular DCFH-DA. Fluorescence intensities were analyzed at 488 nm excitation and 525 nm emission by flow cytometry.

### 2.6. Transient Transfection

The activity of HGF was silenced by transient transfection of siRNA duplexes against HGF (General Biosystems, Anhui, China) with the assistance of a Lipofectamine 2000 reagent (Invitrogen, Carlsbad, California, USA). Cells were treated with oxalate and RSG at 4 h posttransfection, and the expression of related proteins was measured by Western blotting. The sequences of the siRNAs are shown in [Supplementary-material supplementary-material-1].

### 2.7. Renal Oxidative Stress

Lipid peroxidation in whole renal tissue was measured by assaying the malondialdehyde (MDA) content and superoxide dismutase (SOD) enzyme activity according to the manufacturer's protocols (Beyotime Institute of Biotechnology, Shanghai, China) [23].

### 2.8. Ultrasound

We used color ultrasound to detect whether CaOx stones had formed in the rat kidneys. This procedure was performed by experienced sonologists who were blinded to the study design.

### 2.9. Collection and Analysis of Urine and Serum

According to the instructions, creatinine levels in blood and urine were measured with a creatinine assay kit (Wanlei Biological Technology, Shenyang, China). Calcium concentration in urine and serum was measured by an automatic biochemical analyzer (Mindray, China). The content of oxalate in urine is determined by a special detection kit (Trinity Biotech, USA).

### 2.10. Immunofluorescence (IF)

MDCK cells were seeded into 24-well plates and treated as described above. Briefly, the cells were fixed in 4% paraformaldehyde for 30 min, washed with PBS three times for 5 min, and then permeabilized with 0.5% Triton-100 for 60 min. After incubation with primary antibodies overnight at 4°C, the cells were incubated with secondary antibodies in the dark for 60 min and then counterstained with a DAPI nuclear stain for 5 min before images were collected under a fluorescence microscope [24].

### 2.11. Morphological Staining

Kidney tissues were fixed in a formalin solution and then routinely embedded in paraffin. The specimens were subsequently dehydrated and stained with hematoxylin and eosin (H&E). In addition, CaOx crystals were identified by Pizzolato staining.

### 2.12. Immunohistochemistry (IHC)

Briefly, paraffin-embedded renal tissues (4 *μ*m) were immunostained with primary antibodies against p-MET, HGF, and TGF-*β*1 according to the manufacturer's instructions. Image capture and quantification of the stained renal tissues were performed using an Olympus BX51 microscope. Positive cells (brown) were counted using Image-Pro plus 6.0 software.

### 2.13. Western Blot Analysis

MDCK cells exposed to specified concentrations of oxalate and with or without RSG and GW9662 were harvested and homogenized in lysis buffer containing proteinase inhibitors and phosphatase inhibitors (Beyotime Institute of Biotechnology, Shanghai, China). Total protein concentrations of the supernatants were determined with a BCA protein assay (Beyotime Institute of Biotechnology, Shanghai, China). A total of 25 *μ*g of protein was separated by 8% or 10% SDS-PAGE and electrophoretically transferred onto 0.45 *μ*m PVDF membranes. After blocking in 5% nonfat milk for 1 h at room temperature, the membranes were incubated with primary antibodies at 4°C overnight. The membranes were then incubated with a horseradish peroxidase- (HRP-) conjugated secondary antibody for 1 h at room temperature. Finally, the protein bands were visualized with an enhanced chemiluminescence (ECL) Western blotting reagent. The blots were analyzed using Bio-Rad image analysis software, and the results are representative of at least three independent experiments. Proteins from samples from patients with urolithiasis and mice were extracted and homogenized with a homogenizer, and the subsequent steps were the same as above.

### 2.14. Statistical Analysis

Each experiment was performed three times, and all data are expressed as the means ± SD. ANOVA was used to analyze the differences among multiple groups, and a two-tailed *t*-test was used to compare two groups. Values of *p* < 0.05 were considered to indicate a statistically significant difference.

## 3. Results

### 3.1. RSG Promoted the Viability and Proliferation of MDCK Cells Exposed to Oxalate

To determine the effect of RSG on kidney epithelial cells (MDCK cells) treated with oxalate, their viability and proliferation were measured by CCK-8 assays. Consistent with our previous findings, MDCK cell exposure to oxalate led to a prominent concentration-dependent suppression of cell viability. Compared to that in the control group, cell viability was significantly decreased at oxalate concentrations greater than 0.5 mM (data not shown).

**Figure 1:**
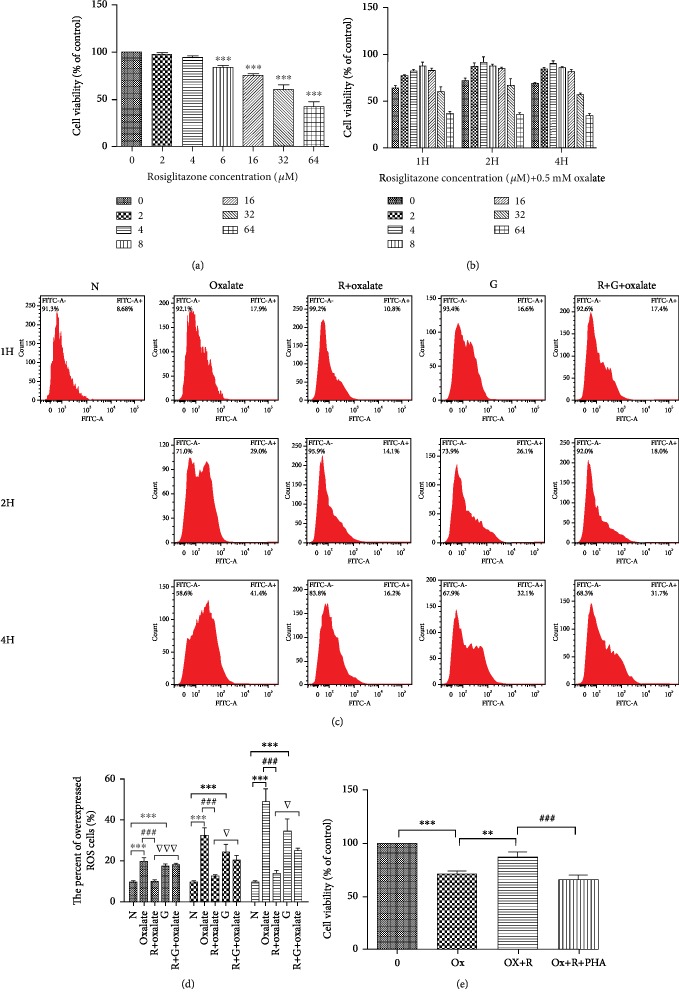
Rosiglitazone (RSG) promoted the viability of MDCK cells. (a) MDCK cells were treated with a concentration gradient of RSG. (b) Cells were cotreated with various concentrations of RSG (0, 2, 4, 8, 16, 32, and 64 *μ*mol/L) and oxalate (0.5 mM) in DMEM medium for 1, 2, and 4 h, and a CCK-8 assay was performed to assess cell viability. (c) The generation of ROS was detected by a DCFH-DA assay. (d) Percent of ROS overexpressing cells per group. (e) Viability of MDCK cells treated with RSG, oxalate, and PHA665752. The data shown are representative of at least three independent experiments. N: normal; G: GW9662; H: hour; PHA: PHA665752. ^∗^*p* < 0.05, ^∗∗^*p* < 0.01, and ^∗∗∗^*p* < 0.001 versus the control group; ^#^*p* < 0.05, ^##^*p* < 0.01, and ^###^*p* < 0.001 versus the 0.5 mM oxalate group; ^▽^*p* < 0.05, ^▽▽^*p* < 0.01, and ^▽▽▽^*p* < 0.001 versus the RSG-treated group.

As shown in Figure 1(a), RSG is not toxic to cells at a range of concentrations. However, RSG inhibited cell viability distinctly at concentrations greater than 8 *μ*mol/L. To determine the function of RSG in oxalate-induced MDCK cell injury, we treated MDCK cells with gradient concentrations of RSG and 0.5 mM oxalate for 1, 2, or 4 h. Importantly, peak protection occurred after exposure to an RSG concentration of 4 *μ*M for 2 and 4 h; nevertheless, treatment with 5 *μ*M or higher RSG for 2 h significantly inhibited MDCK cell viability, as presented in Figure 1(b). Therefore, incubation with 4 *μ*M RSG and 0.5 mM oxalate for 2 h was used as the treatment condition in the subsequent experiments.

### 3.2. RSG Provides Protection against Oxalate-Induced Oxidative Damage to MDCK Cells

**Figure 2:**
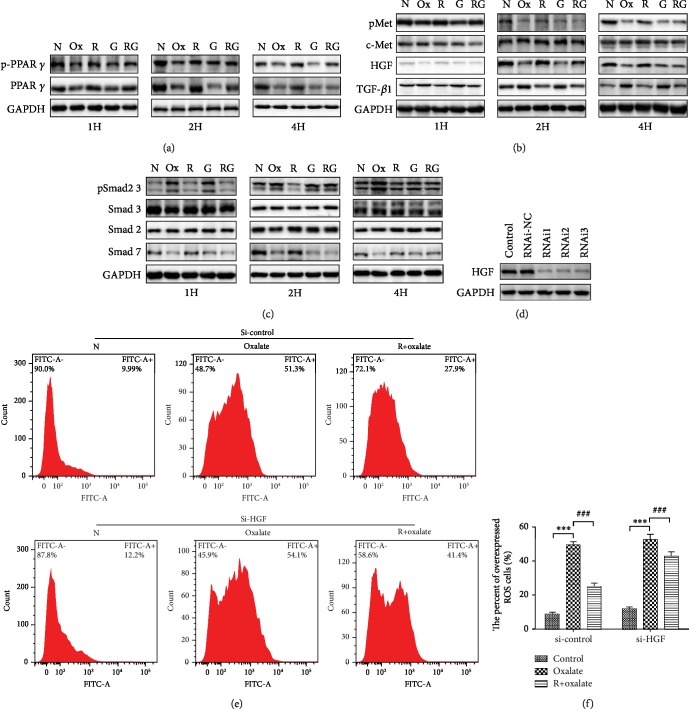
RSG promoted the activity of MDCK cells by inhibiting TGF-*β*1/Smad and activating the HGF/c-Met pathway. (a–c) Protein expression levels of TGF-*β*1, HGF, c-Met, p-Met, PPAR-*γ*, p-PPAR-*γ*, Smad7, Smad2, Smad3, and pSmad2 3 were analyzed using Western blotting after treatment for 1, 2, and 4 hours. The relative protein levels were normalized to the GAPDH level. (d) Western blot assay of HGF after siRNA transfection. (e, f) siRNA-mediated knockdown of HGF led to significant inhibition of the antioxidant capacity of RSG. The quantitative results are expressed as the means ± SD of three experiments. N: normal; Ox: oxalate; R: rosiglitazone; G: GW9662; H: hour. ^∗∗∗^*p* < 0.001 versus the control group; ^###^*p* < 0.001 versus the 0.5 mM oxalate group.

Cumulative evidence has demonstrated that oxidative stress is closely related to oxalate-induced renal epithelial cell damage. To assess whether RSG protects MDCK cells from oxidative stress induced by sodium oxalate treatment for different durations (1, 2, and 4 h), intracellular ROS were visualized by flow cytometry, which measured the generation of fluorescent 2,7-dichlorofluorescein diacetate (DCFH-DA) in MDCK cells. As shown in Figure 1(c), ROS generation in response to oxalate was significantly increased versus that in untreated controls in a concentration- and time-dependent manner. However, ROS generation was markedly blunted compared to that in the oxalate-treated group in the presence of RSG (*p* < 0.05). In addition, the PPAR-*γ* inhibitor GW9662, PHA665752 (c-Met inhibitor), and siRNA silencing of HGF markedly abolished the protective effect of RSG (Figures 1(e), 2(e), and 2(f)). Considering the effect of siRNA knockdown of HGF, RNAi1 was selected for knockout in subsequent experiments (Figure 2(d)).

### 3.3. The Effect of RSG on PPAR-*γ* Expression

**Figure 3:**
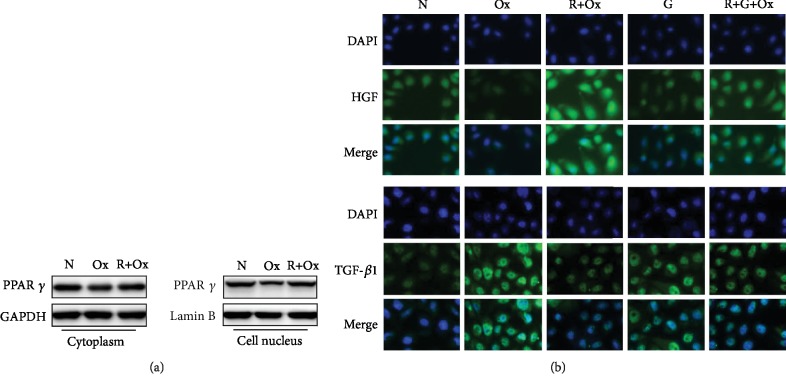
(a) The expression of PPAR-*γ* in cytoplasm and nucleus was detected by Western blotting *in vitro.* (b) Representative fluorescence images of HGF and TGF-*β*1 expression were evaluated (×200). N: normal; Ox: oxalate; R: rosiglitazone; G: GW9662.

**Figure 4:**
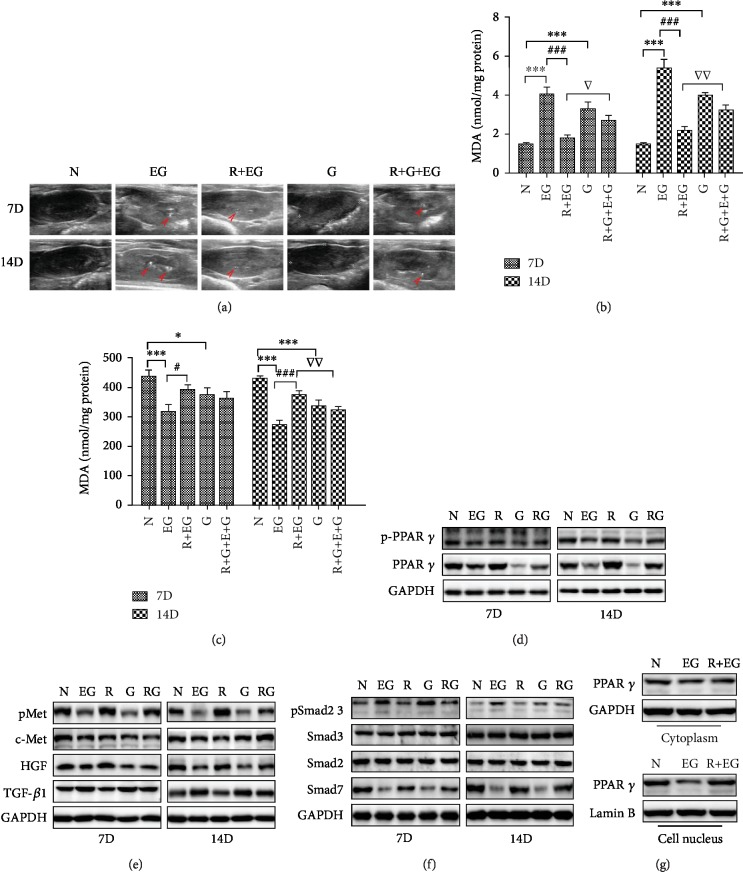
(a) The formation of calcium oxalate crystals in different groups was observed by color Doppler ultrasound (red arrow indicates the stone). (b, c) Effect of RSG on the MDA level and SOD activity in renal tissue on days 7 and 14, respectively. (d–f) Protein expression *in vivo* was detected by Western blotting. (g) The expression of PPAR-*γ* in cytoplasm and nucleus was detected by Western blotting *in vivo*. The data shown are representative of at least three independent experiments. N: normal; EG: ethylene glycol; R: rosiglitazone; G: GW9662; D: day. ^∗^*p* < 0.05 and ^∗∗∗^*p* < 0.001 versus the control group; ^#^*p* < 0.05 and ^###^*p* < 0.001 versus EG group; ^▽^*p* < 0.05 and ^▽▽^*p* < 0.01 versus the RSG-treated group.

To explore the mechanism by which RSG exerts renal protection, we first examined the influence of oxalate on PPAR-*γ* and p-PPAR-*γ* (Ser112) expression. Our data have shown that the expression of PPAR-*γ* and p-PPAR-*γ* (Ser112) was detected in renal epithelial cells both *in vitro* and *in vivo*. However, oxalate treatment could result in a decrease in PPAR-*γ* expression in both the nucleus and cytoplasm in MDCK cells. PPAR-*γ* and p-PPAR-*γ* (Ser112) were also decreased in the EG group compared with the normal group. In contrast to oxalate-treated cells, the expression of the PPAR-*γ* protein increased in the presence of RSG in both the nucleus and cytoplasm in MDCK cells, and increased PPAR-*γ* and p-PPAR-*γ* (Ser112) expression was also observed *in vivo*. In addition, the PPAR-*γ* inhibitor GW9662 could simulate the effect of oxalate on PPAR-*γ* expression (Figures 2(a), 3(a), 4(d), and 4(g); Figures [Supplementary-material supplementary-material-1] and [Supplementary-material supplementary-material-1]).

### 3.4. The Effect of RSG on HGF/c-Met and the TGF-*β*1/Smad Pathway

**Figure 5:**
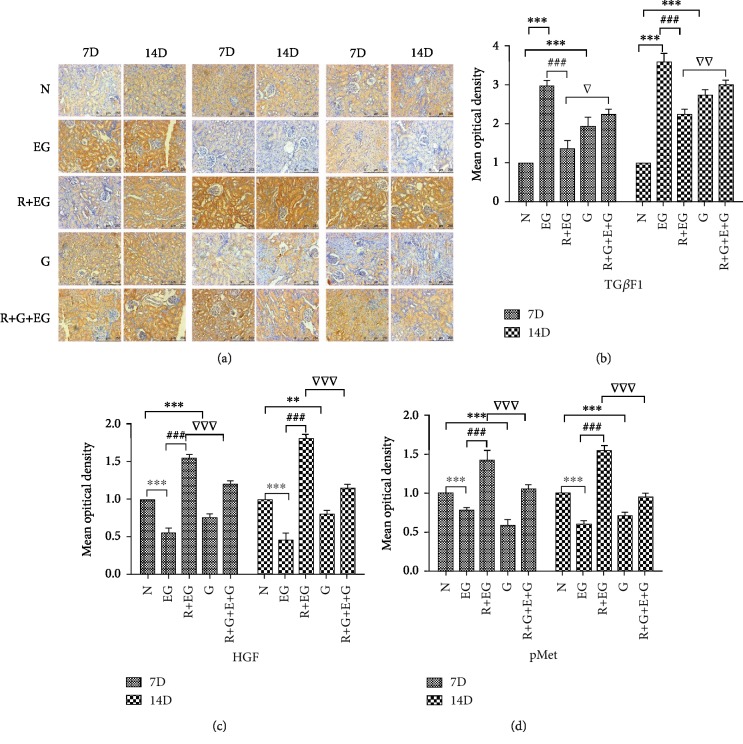
Representative immunohistochemistry images of kidney tissues. (a) Level of TGF-*β*1 was decreased in the RSG-treated group, while accumulation of HGF and p-Met by IHC staining in the RSG group was higher than that in the EG-induced rat model (×200). (b–d) Immunohistochemical expressions of TGF-*β*1, HGF, and p-Met were evaluated by densitometric analysis. The quantitative results are expressed as the means ± SD of three experiments. N: normal; EG: ethylene glycol; R: rosiglitazone; G: GW9662; D: day. ^∗^*p* < 0.05, ^∗∗^*p* < 0.01, and ^∗∗∗^*p* < 0.001 versus the control group; ^###^*p* < 0.001 versus EG group; ^▽^*p* < 0.05, ^▽▽^*p* < 0.01, and ^▽▽▽^*p* < 0.001 versus the RSG-treated group.

PPAR-*γ* is upstream of the HGF/c-Met signaling pathway and can regulate the activation of Smad7; therefore, we determined whether PPAR-*γ* regulates the expression of HGF, c-Met, and Smad7 in renal tubular epithelial cells during kidney stone formation. Western blotting, IF, and IHC were used to determine the expression of HGF, c-Met, Smad7, and PPAR-*γ*. As shown in Figures 2–5, compared to that in the oxalate groups, the expression of HGF, p-Met, and Smad7 was upregulated in the RSG treatment groups. However, we found that cotreatment with GW9962, an effective PPAR-*γ* inhibitor, significantly reversed the overexpression of HGF, p-Met, and Smad7. Furthermore, compared to that in the control group, the expression of TGF-*β*1 and phosphorylated Smad2 3 increased in a time-dependent manner in the groups induced with either oxalate or EG. In contrast, in both animal and cell experiments, the expression of the abovementioned proteins was conspicuously reduced in a time-dependent manner in response to RSG.

### 3.5. Assessment of Renal Function and Calcium and Oxalate Metabolism

To verify the protective effect of RSG on renal function and calcium and oxalate metabolism, the contents of creatinine, calcium, and oxalate in the urine and serum were measured on days 7 and 14. As shown in Tables 1 and 2, compared with those in the normal group, creatinine and oxalate levels in the EG group increased tremendously at 7 and 14 days (*p* < 0.001), while RSG treatment significantly ameliorated renal function damage caused by CaOx crystals and reduced the increase of oxalate. However, the addition of GW9662 could inhibit the renal protective effect of RSG. In addition, the calcium concentration in the serum and urine of the EG group was higher than that in the normal group, and there was no significant difference among the other groups.

### 3.6. RSG Attenuated EG-Induced Oxidative Stress *In Vivo*

To further evaluate the role of RSG in antioxidant stress in animal experiments, SOD and MDA were measured to determine the level of lipid peroxidation. As presented in Figure 4(c), the SOD level was significantly higher in the EG+RSG treatment group than in the EG treatment group on days 7 and 14. In contrast, compared to the control conditions, exposing the kidney tissues to EG dramatically blunted SOD activity in a dose-dependent manner. In addition, no remarkable variation was observed in the SOD levels between the EG+RSG+GW9662 group and the RSG+EG group on day 7. However, as shown in Figure 4(b), compared to the SOD activity, the MDA levels showed opposite effects in the corresponding groups.

### 3.7. RSG Markedly Ameliorated the Deposition of CaOx Crystals and EG-Induced Histopathological Morphological Alterations in the Rat Model

**Figure 6:**
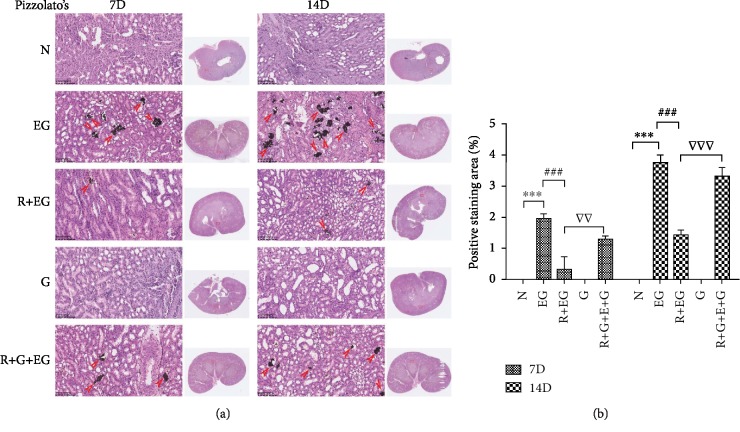
(a) Pizzolato's method was performed to observe the formation of renal crystal deposits at the 7th day and 14th day time points (×200). (b) The semiquantitative analysis bar graph was used to evaluate the deposition of CaOx. Each value represents the mean ± SD for triplicate samples. The red arrows indicate CaOx crystals. N: normal; EG: ethylene glycol; R: rosiglitazone; G: GW9662; D: day. ^∗∗∗^*p* < 0.001 versus the control group; ^###^*p* < 0.001 versus the EG group; ^▽^*p* < 0.05 and ^▽▽▽^*p* < 0.001 versus the RSG-treated group.

To determine whether RSG could suppress the formation of kidney stones, Pizzolato staining and color Doppler ultrasound were performed to observe the formation of renal crystal deposits. Crystal deposits were clearly observed in the distal renal tubule at the junction of the renal cortex and medulla (Figure 6(a); [Supplementary-material supplementary-material-1]). The representative images showing crystallization in the kidneys are consistent with the color ultrasound images, as illustrated in Figure 4(a). Compared to EG treatment alone, RSG resulted in a significant reduction in the retention of CaOx crystals. Histopathologic examination revealed evident atrophy of glomeruli and renal tubular epithelial cell swelling and deformation in EG-treated rats. However, the characteristic morphological changes of hyperoxaluric rats in the presence of RSG were significantly attenuated ([Supplementary-material supplementary-material-1]). The degree and quantity of dark, dense CaOx crystals increased substantially over time in the EG-only group, and this increase was significantly reduced after RSG injection. However, the potential antinephrolithic role of RSG was reversed by GW9662 administration, and no positive staining areas were found for the control group or the GW9662 treatment group after microscopic observation.

## 4. Discussion

**Figure 7:**
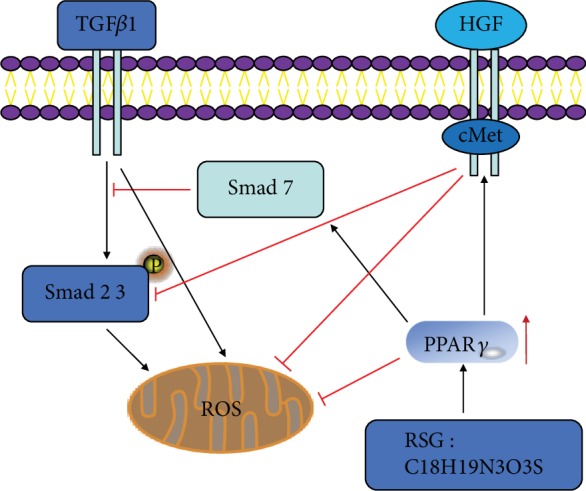
Diagram illustrating the signaling pathways involved in RSG inhibiting the formation of kidney stones.

The present study demonstrates the significant role of the PPAR-*γ*-HGF/c-Met and PPAR-*γ*-TGF-*β*1/Smad pathways in both *in vivo* and *in vitro* CaOx nephrolithiasis models. Oxidative stress plays a critical role in the pathogenesis of CaOx nephrolithiasis [25]. We found that oxalate antagonized the expression and activity of PPAR-*γ* and promoted ROS by increasing TGF-*β*1/Smad signaling and decreasing HGF/c-Met signaling. Moreover, we provide evidence that RSG, a direct target of PPAR-*γ*, suppresses oxalate-induced ROS by inactivating the TGF-*β*1/Smad signaling pathway and activating the HGF/c-Met signaling pathway. Collectively, the current results implicate the decreasing expression and activity of PPAR-*γ* as mechanisms that initiate CaOx nephrolithiasis. Taken together, our data suggest that PPAR-*γ* activation could be an effective method to protect against oxalate-mediated ROS by enhancing HGF/c-Met signaling and suppressing TGF-*β*1/Smad signaling in renal tubular cells (Figure 7).

In the kidney, PPAR-*γ* is mainly selectively expressed in the medullary collecting ducts and pelvic urothelium and is significant in the pathogenesis of many kinds of kidney diseases [26]. However, the effect of oxalate on PPAR-*γ* expression and activity and the downstream consequences have not been defined in renal tubular epithelial cells. We found that oxalate targets PPAR-*γ* and promotes ROS production in tubular epithelial cells and a rat model. Our results demonstrate that PPAR-*γ* is present in tubular cells and is downregulated upon oxalate treatment. Oxalate inhibits not only PPAR-*γ* expression but also phosphorylation, which indicates PPAR-*γ* activity. Our results are consistent with some reports demonstrating that in animal models of diabetes, PPAR-*γ* mRNA and protein levels are significantly reduced, while superoxide radical levels are increased [27, 28]. Administration of the PPAR-*γ* antagonist GW9662 has been shown to increase ROS concentrations in some cell types [29]. These findings provide evidence that PPAR-*γ* is a critical intermediate modulator that is beneficial in reducing oxidative stress. The PPAR-*γ* agonist RSG has been reported to have anti-inflammatory and antioxidative effects. We found that RSG treatment suppressed ROS production both *in vitro* and *in vivo*. These results are in accordance with some reports suggesting that the PPAR-*γ* agonist PGZ suppressed renal crystal formation as well as acute renal injury by attenuating oxidative stress, apoptosis, and inflammatory response in rat models [25]. To further elucidate the important role of PPAR-*γ*, we inhibited PPAR-*γ* using GW9662. Our results showed that GW9662 significantly inhibited PPAR-*γ* expression and increased intracellular ROS levels. Thus, our current study demonstrates that oxalate increases ROS levels by suppressing PPAR-*γ* in tubular epithelial cells and that GW9662 completely blocks the protective effect of RSG, which indicates that RSG plays a protective role by activating PPAR-*γ*.

Oxalate-induced molecular changes are a major contributor to ROS production and the development of CaOx nephrolithiasis [30]. PPAR-*γ* has thus become a therapeutic target for diabetic nephropathy, hypertensive nephropathy, glomerulonephritis, and other chronic kidney diseases [5, 28]. PPAR-*γ* agonists have been increasingly recognized as possessing potent antifibrotic activity and antioxidative effects that effectively prevent renal dysfunction resulting from chronic kidney diseases [21]. Treatment with the PPAR-*γ* agonist PGZ can relieve oxidative stress and exert nephroprotective effects against cisplatin-induced renal damage [31]. Our experiment clearly shows that RSG can effectively inhibit increases in MDA concentrations and decreases in SOD levels in a hyperoxaluric rat model. In an *in vitro* experiment, we also observed that RSG significantly alleviated the oxalate-induced ROS level increase in MDCK cells and that RSG effectively suppressed renal crystal formation in the rat model. In addition, as some ligands of PPAR-*γ* have functions independent of PPAR-*γ*, we used GW9662 to test whether the effect of RSG is PPAR-*γ* dependent [32]. We found that GW9662 nearly completely blocks the protective effect of RSG, which indicates that RSG plays a protective role by activating PPAR-*γ*. Similar results were obtained using other PPAR agonists, such as PGZ, TGZ, and 15d-PGJ2. Thus, we found that PPAR-*γ* activation plays an important role in both hyperoxaluria-induced renal injury *in vivo* and in oxalate-induced cellular ROS accumulation in MDCK cell cultures *in vitro*.

Although our above findings demonstrated that PPAR-*γ* plays a critical role in maintaining redox status, the downstream consequences of PPAR-*γ* in the presence of oxalate have not been explored. The major finding of this study is that endothelial PPAR-*γ* protects tubular cells from ROS damage by regulating the balance of TGF-*β*1/Smad and HGF/c-Met signaling. The increase in TGF-*β*1 expression is closely correlated with oxalate-induced tubular epithelial cell injury and the development of nephrolithiasis. One of the proposed mechanisms is the antagonistic interaction between PPAR-*γ* and TGF-*β* signaling. PPAR-*γ* agonists have been reported to exert antifibrotic effects in human proximal tubule cells and to reduce high glucose levels by attenuating TGF-*β*1 [31]. PPAR-*γ* can inhibit the phosphorylation of Smad2 and Smad3 and repress the TGF-*β*1 signaling pathway [33]. PPAR-*γ* activation by its agonists, such as RSG, PGZ, and 15d-PGJ2, or genetic PPAR-*γ* overexpression prevented the induction of ROS induced by TGF-*β*1 [31]. However, some cytokines and chemokines have been shown to regulate PPAR-*γ* expression. TGF-*β*1 reduces PPAR-*γ* expression in fibroblasts and hepatic stellate cells [34]. Moreover, TGF-*β*1-induced ROS were reported to suppress PPAR-*γ* expression and activity by enhancing oxidative stress damage, and activated PPAR-*γ*, in turn, protects renal tubular cells by inhibiting the oxidative response [35]. Consistent with these previous findings, in our study, rat tissues exhibited significantly enhanced expression and activity of TGF-*β*1, Smad2, and Smad3 after 7 days of treatment with EG, but TGF-*β*1 overexpression related to renal injury induced by hyperoxaluria was prevented by RSG injection in the rat model. Similar results were observed *in vitro*. These suppressive effects of RSG on TGF-*β*1 in the kidneys were reversed by GW9662. Our results clearly illustrated the antagonistic interaction between PPAR-*γ* and the TGF-*β*1/ROS axis in renal tubular cells.

HGF, as a heparin-binding disulfide-linked heterodimer, is a potent antifibrotic cytokine and plays an antifibrotic role in a variety of organs [18]. HGF and its specific receptor c-Met have multiple biological functions in renal tubular cells, such as antifibrotic and antioxidation effects [36]. HGF exerts remarkable antioxidative effects by promoting ROS scavenging and reducing ROS production and accumulation in rat mesangial cells [37]. Since HGF is an endogenous secreted cytokine, the best way to increase HGF expression *in vivo* is to activate its upstream molecules and then upregulate its expression. However, the detailed mechanism of HGF secretion from renal tubular cells remains unclear. Recent studies revealed that HGF acts as a downstream effector of PPAR-*γ* agonists. Transfection studies revealed that 15d-PGJ2 (a PPAR-*γ* agonist) stimulated the activity of the HGF gene promoter due to the presence of a novel PPRE, and PPAR-*γ* agonists promoted not only HGF expression but also c-Met phosphorylation in mesangial cells [18]. The PPAR-*γ* agonists telmisartan and irbesartan play an antirenal fibrosis role by increasing HGF production and secretion [27, 38]. Recently, the protective effect of PPAR-*γ* agonists was considered partly to be the result of increasing HGF expression [39]. Therefore, we postulate that the PPAR-*γ*-HGF/c-Met axis also exists in tubular cells. In this study, after oxalate treatment, we found a decrease in HGF and c-Met expression in renal tubular cells following repression of PPAR-*γ*. These phenomena could be reversed by the addition of RSG. Moreover, treatment of MDCK cells with GW9662 alone decreased HGF and c-Met expression as well as c-Met phosphorylation. Combined with previous research by others, our results demonstrate that a constitutively activated PPAR-*γ*-HGF/c-Met axis exists in tubular cells and exerts nephroprotective effects. Next, we found that GW9662 abolished RSG-induced HGF/c-Met expression in response to oxalate, and the protective effect of RSG could be eliminated by a c-Met inhibitor (PHA665752) or siRNA silencing of HGF. These results indicated that the nephroprotective role of RSG largely depends on the PPAR-*γ*-HGF/c-Met axis.

## 5. Conclusions

In summary, we suggest that oxalate inhibits PPAR-*γ* expression and activity via the TGF-*β*1-ROS pathway in renal tubular cells and that enhanced TGF-*β*1 expression is a result of PPAR-*γ* reduction. Furthermore, the repression of PPAR-*γ* also downregulates the HGF/c-Met pathway. The imbalance between TGF-*β*1 and HGF/c-Met leads to the accumulation of ROS and related protein damage, which becomes the initial event in the development of nephrolithiasis. PPAR-*γ* agonists not only inhibit TGF-*β*1 signaling but also enhance the HGF/c-Met pathway in tubular cells exposed to oxalate and exert antioxidant and nephroprotective effects. Therefore, activation of PPAR-*γ* may be a potent target for protecting tubular cells from hyperoxaluria-induced oxidative injury and inhibiting CaOx crystal deposition. We believe that new PPAR-*γ* agonists with fewer side effects that emerge in the future may be a therapy for nephrolithiasis.

## Figures and Tables

**Table 1:** Comparison of the contents of creatinine among groups.

	N	EG	R+EG	G	R+G+EG
*Serum 7D*					
Creatinine (mg/dL)	1.48 ± 0.02	1.94 ± 0.07^a^^∗∗∗^	1.70 ± 0.05^b^^∗∗^	1.58 ± 0.01	1.76 ± 0.03
*Serum 14D*					
Creatinine (mg/dL)	1.55 ± 0.71	2.36 ± 0.04^a^^∗∗∗^	1.66 ± 0.10^b^^∗∗∗^	1.58 ± 0.06	2.00 ± 0.05^c^^∗∗∗^
*Urine 7D*					
Creatinine (mg/dL)	102.34 ± 5.20	143.15 ± 1.30^a^^∗∗∗^	125.05 ± 4.83^b^^∗∗^	110.78 ± 0.44	129.21 ± 4.55
*Urine 14D*					
Creatinine (mg/dL)	107.67 ± 7.35	223.12 ± 7.96^a^^∗∗∗^	167.60 ± 6.86^b^^∗∗∗^	136.12 ± 8.99^a^^∗^	196.85 ± 8.99^c^^∗^

^a^^∗∗∗^ *p* < 0.001 versus the control group; ^b^^∗∗^*p* < 0.01 and ^b^^∗∗∗^*p* < 0.001 versus EG group; ^c^^∗^*p* < 0.01 and ^c^^∗∗∗^*p* < 0.001 versus the RSG-treated group.

**Table 2:** Comparison of calcium and oxalate metabolism levels among groups.

	N	EG	R+EG	G	R+G+EG
*Serum 7D*					
Ca (mmol/L)	2.42 ± 0.03	2.50 ± 0.03^a^^∗^	2.46 ± 0.01	2.43 ± 0.02	2.48 ± 0.02
*Serum 14D*					
Ca (mmol/L)	2.41 ± 0.03	2.51 ± 0.02^a^^∗^	2.45 ± 0.02	2.42 ± 0.02	2.46 ± 0.02
*Urine 7D*					
Ca (mg/day)	2.50 ± 0.03	2.59 ± 0.02^a^^∗^	2.56 ± 0.02	2.45 ± 0.02	2.57 ± 0.01
*Urine 14D*					
Ca (mg/day)	2.49 ± 0.01	2.62 ± 0.03^a^^∗∗∗^	2.57 ± 0.03	2.46 ± 0.01	2.61 ± 0.02
*Urine 7D*					
Oxalate (mg/day)	1.54 ± 0.04	4.27 ± 0.21^a^^∗∗∗^	3.05 ± 0.22^b^^∗∗∗^	1.57 ± 0.03	3.63 ± 0.21^c^^∗∗∗^
*Urine 14D*					
Oxalate (mg/day)	1.56 ± 0.03	8.33 ± 0.29^a^^∗∗∗^	5.01 ± 0.19^b^^∗∗∗^	1.56 ± 0.03	5.75 ± 0.20^c^^∗∗∗^

^a^^∗^ *p* < 0.05 and ^a^^∗∗∗^*p* < 0.001 versus the control group; ^b^^∗∗∗^*p* < 0.001 versus EG group; ^c^^∗∗∗^*p* < 0.001 versus the RSG-treated group.

## Data Availability

The data used to support the findings of this study are available from the corresponding author upon request.

## Supplementary Materials

Supplementary Materials Table S1: the siRNA sequences for the HGF gene. Figure S1: the expression of related proteins *in vitro* is indicated by a graphical representation (A-H). The expression level of PPAR-*γ* in the cytoplasm and nucleus *in vitro* is illustrated by a graphical representation (I). Figure S2: the expression of proteins *in vivo* is illustrated by a graphical representation (A-H). The expression level of PPAR-*γ* in the cytoplasm and nucleus *in vivo* is illustrated by a graphical representation (I). Figure S3: black-stained crystals in the whole cross-section were observed in original pictures of kidney Pizzolato staining under a 10-fold magnification. Figure S4: histopathological and morphological changes of the kidney were observed in different groups under a 200-fold magnification.
