# Supplementary material for: Rosiglitazone Suppresses Calcium Oxalate Crystal Binding and Oxalate-Induced Oxidative Stress in Renal Epithelial Cells by Promoting PPAR-γ Activation and Subsequent Regulation of TGF-β1 and HGF Expression
Source: Oxid Med Cell Longev. 2019 Nov 12;2019:4826525. doi: 10.1155/2019/4826525 (PMC6875173; doi:10.1155/2019/4826525)
Supplement: Supplementary Materials — Table S1: the siRNA sequences for the HGF gene. Figure S1: the expression of related proteins in vitro is indicated by a graphical representation (A-H). The expression level of PPAR-γ in the cytoplasm and nucleus in vitro is illustrated by a graphical representation (I). Figure S2: the expression of proteins in vivo is illustrated by a graphical representation (A-H). The expression level of PPAR-γ in the cytoplasm and nucleus in vivo is illustrated by a graphical representation (I). Figure S3: black-stained crystals in the whole cross-section were observed in original pictures of kidney Pizzolato staining under a 10-fold magnification. Figure S4: histopathological and morphological changes of the kidney were observed in different groups under a 200-fold magnification. [file 4826525.f1.pdf]

Additional files

Table S1. The siRNA sequences for the HGF gene.

siRNA	Sense (5’-3’)
Si-NC (random control sequence)	UUCUCCGAACGUGUCACGUTT
si-HGF-1	GUAAAGACCUACAGGAAAATT
si-HGF-2	CAUGACAAGUGGAGUGAAATT
si-HGF-3	GGGUAAAGACCUACAGGAATT

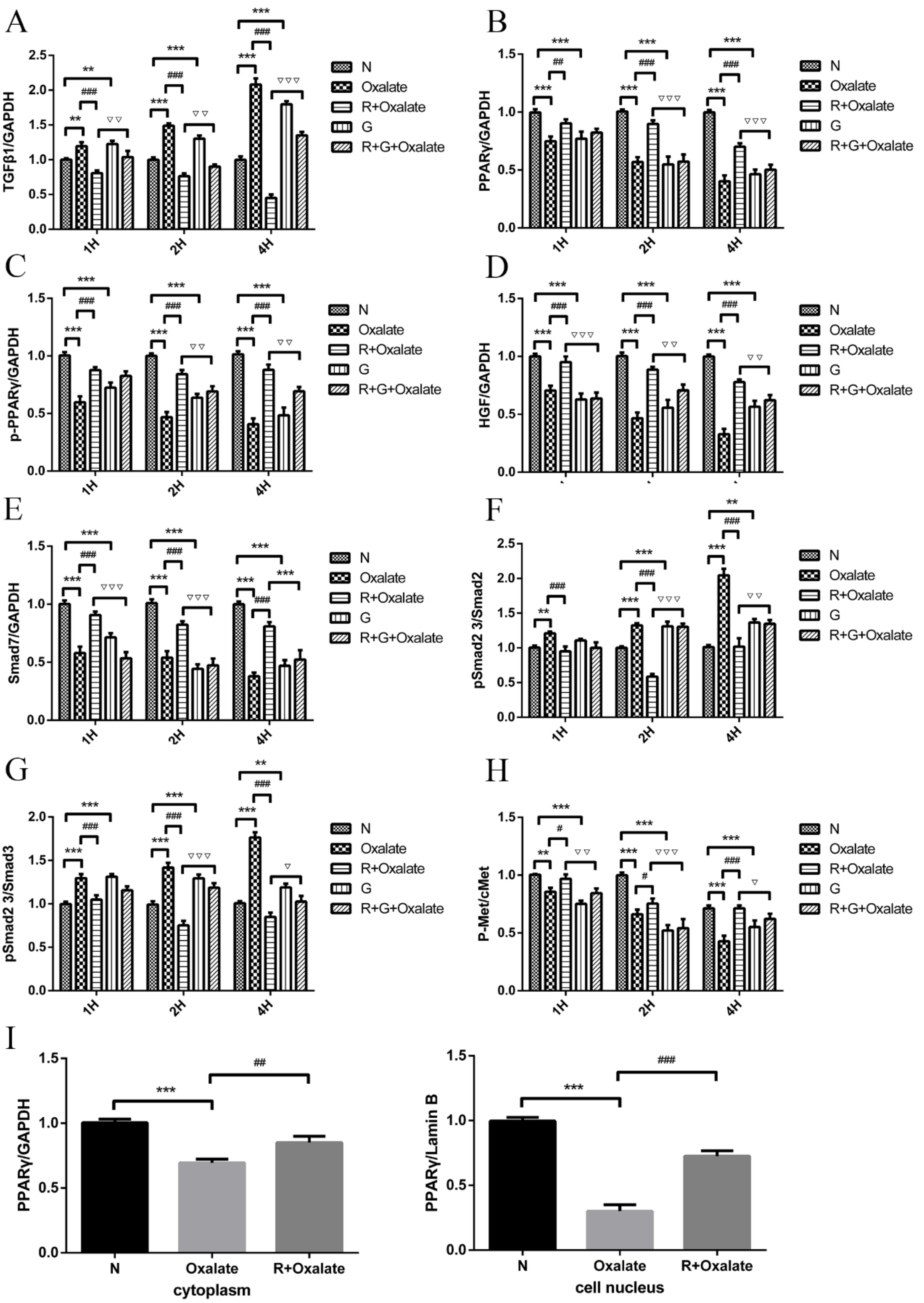

Figure S1**.** The expression of related proteins *in vitro* was indicated by graphical representation (A-H). The expression level of PPAR-γ in cytoplasm and nucleus *in vitro* was illustrated by graphical representation (I). N: normal, R: rosiglitazone, G: GW9662, H: hour. $^{**}p < 0.01$ and $^{***}p < 0.001$ versus the control group; $^{\#}p < 0.05$ and $^{\#\#\#}p < 0.001$ versus the 0.5 mM oxalate group; $^{\triangledown}p < 0.05$, $^{\triangledown\triangledown}p < 0.01$ and $^{\triangledown\triangledown\triangledown}p < 0.001$ versus the RSG-treated group.

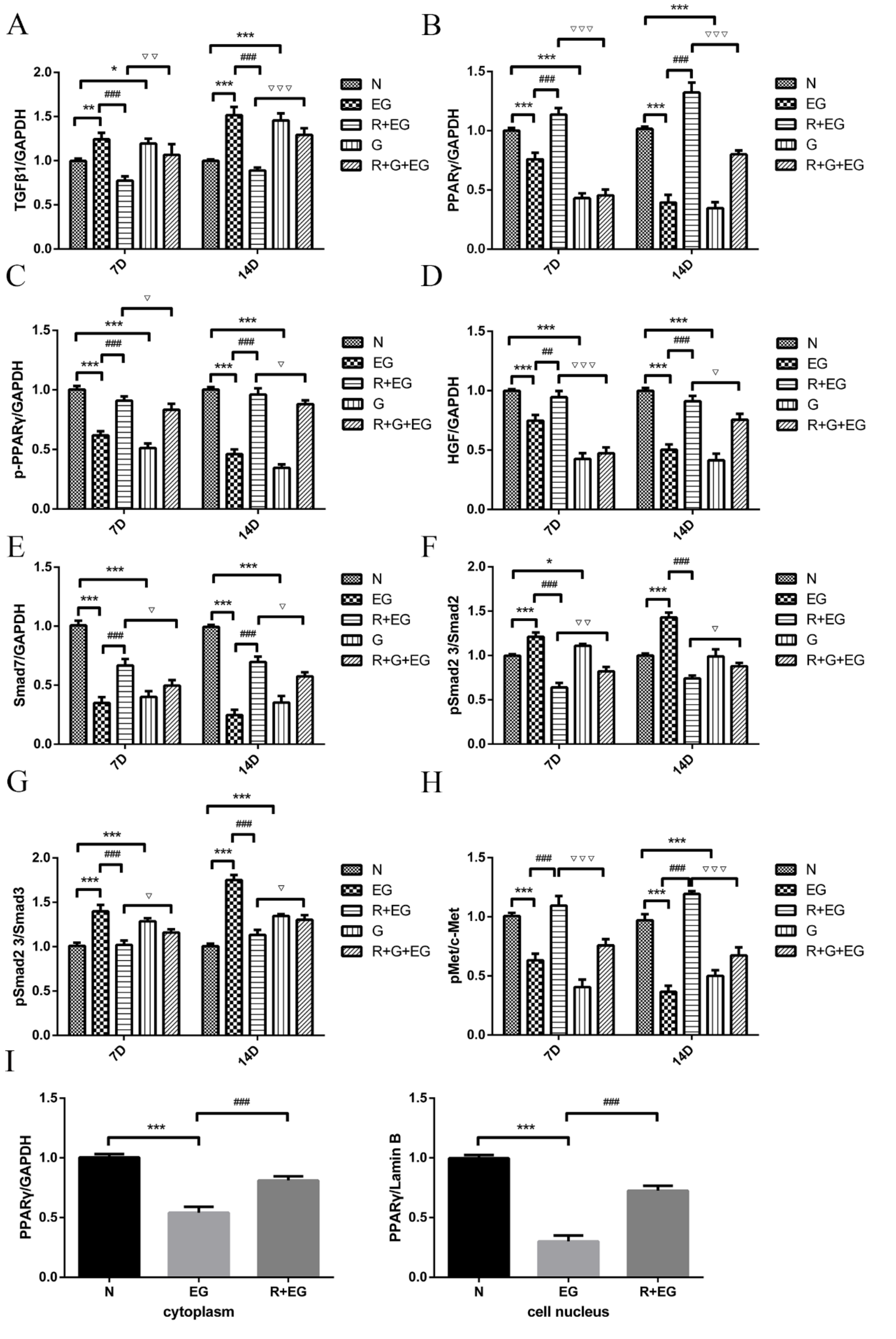

Figure S2. The expression of proteins *in vivo* was displayed by graphical representation (A-H). The expression level of PPAR-γ in cytoplasm and nucleus *in vivo* was illustrated by graphical representation (I). N: normal, EG: ethylene glycol, R: rosiglitazone, G: GW9662, H: hour. $^{*}p < 0.05$, $^{**}p < 0.01$ and $^{***}p$

< 0.001 versus the control group; $^{\#\#}p < 0.01$, and $^{\#\#\#}p < 0.001$ versus EG group; $^{\triangledown}p < 0.05$, $^{\triangledown\triangledown}p < 0.01$ and $^{\triangledown\triangledown\triangledown}p < 0.001$ versus the RSG-treated group.

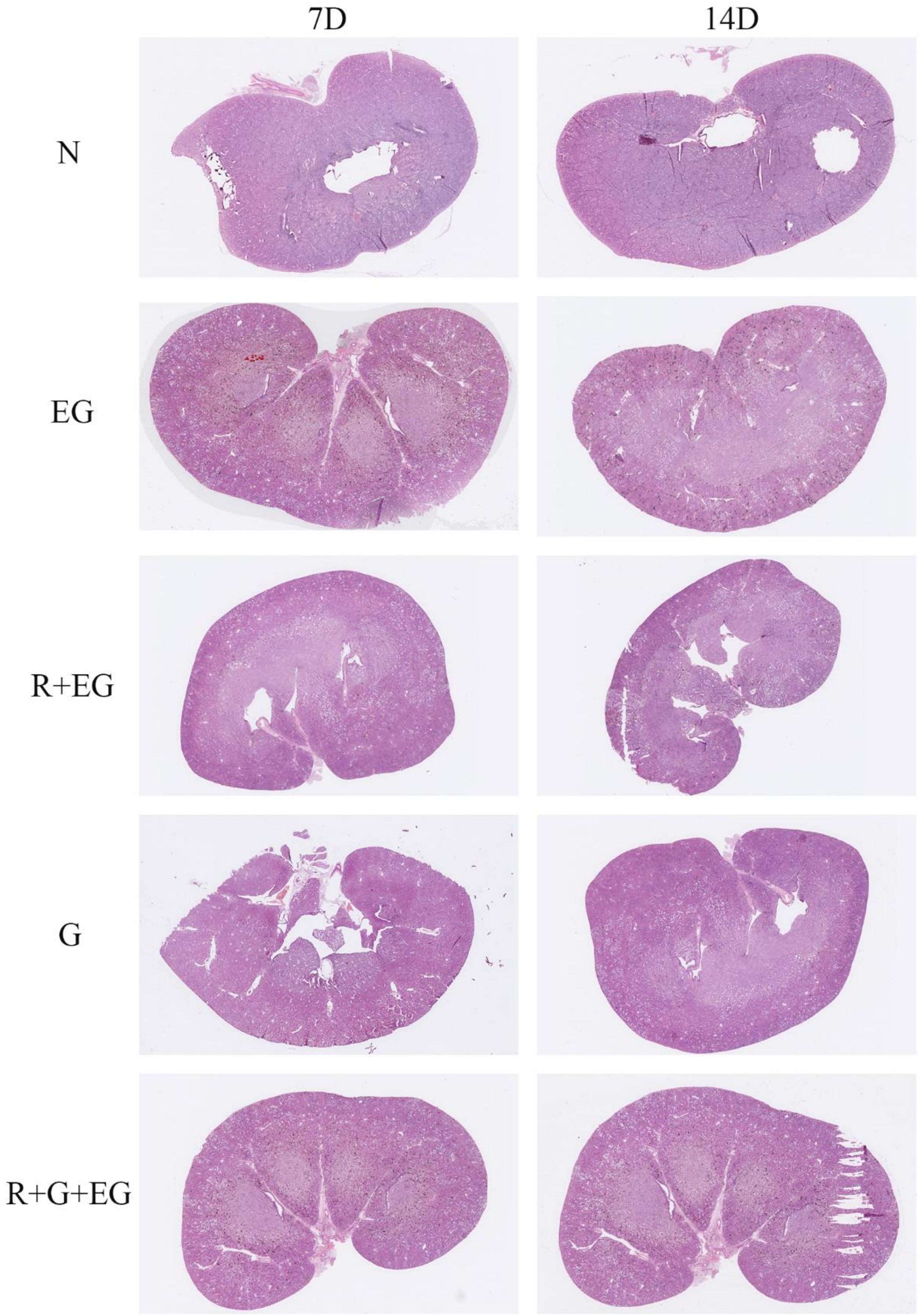

Figure S3. Black stained crystals in the whole cross-section were observed in original pictures of kidney Pizzolato staining under 10-fold magnification field.

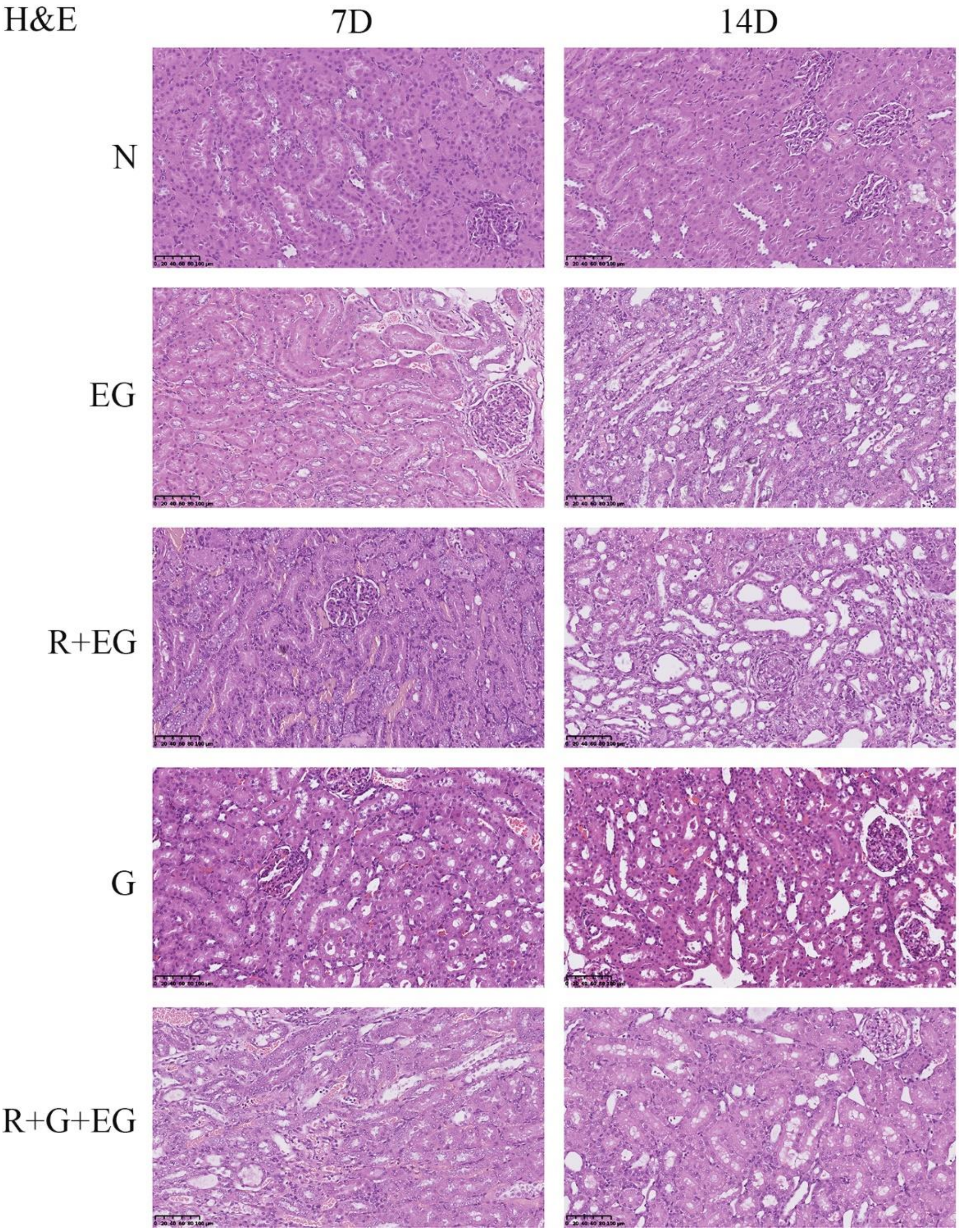

Figure S4. Histopathological and morphological changes of kidney were observed in different groups under a 200-fold magnification.
